# Supplementary figures and images for: HDAC6/aggresome processing pathway importance for inflammasome formation is context-dependent
Source: J Biol Chem. 2024 Jan 8;300(2):105638. doi: 10.1016/j.jbc.2024.105638 (PMC10850954; doi:10.1016/j.jbc.2024.105638)

Fig. S1

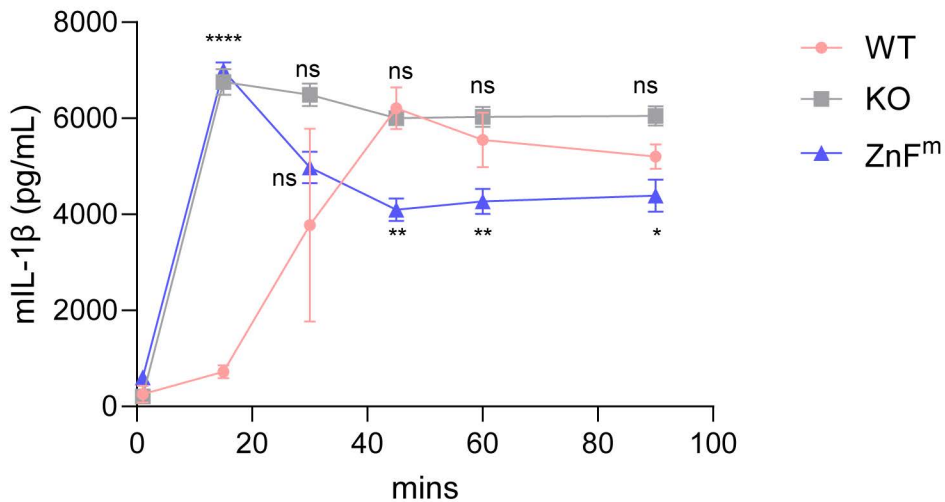

Fig.S2

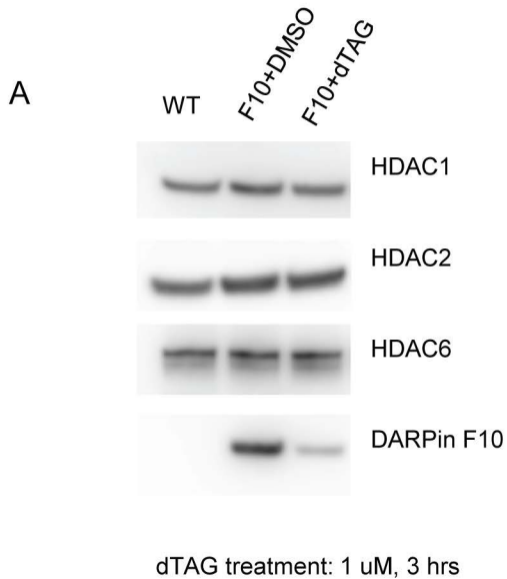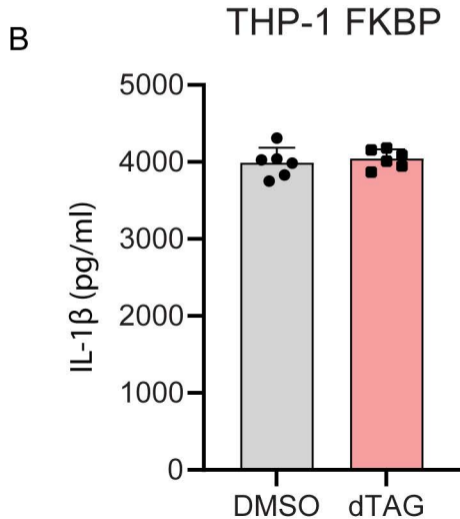

Fig.S3

A

### 3 day CTG assay for XY-07-035

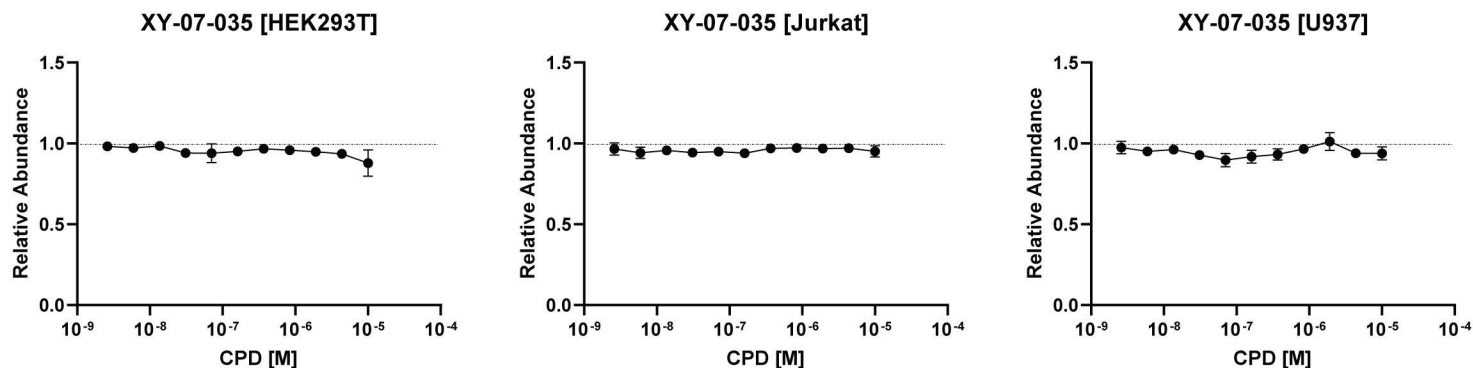

5000 cells / well, 70 hours treatment

B

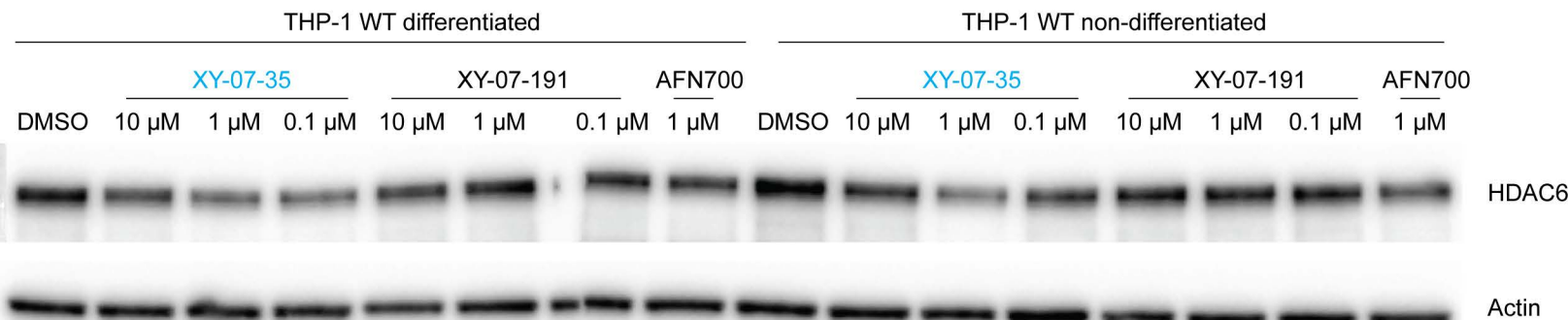

Fig. S4

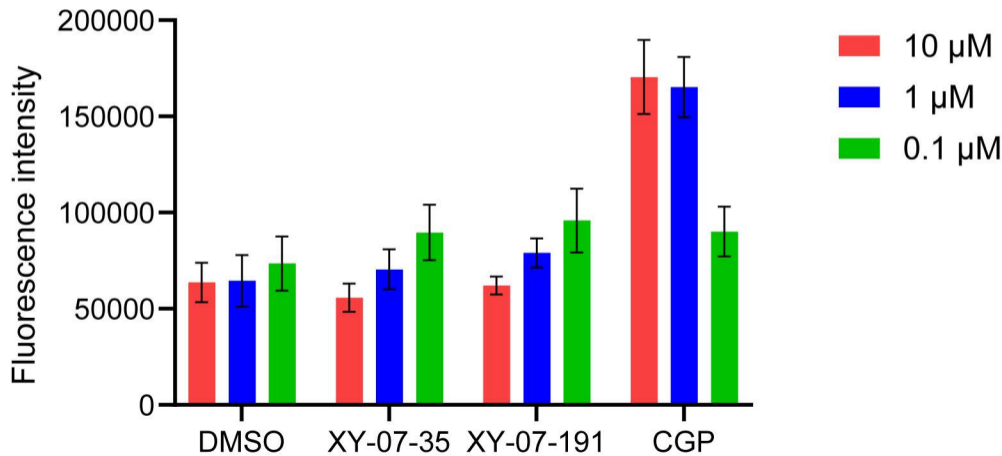

Supplement: Supporting Figure S1 — BMDM cells were primed by LPS (1 μg/ml) for 4 hrs and then challenged with 20 μM nigericin. 50 μl supernatant was collected every 15 min (up to 90 min) and mIL-1β level was determined by HTRF assay. Two-Way ANOVA test was used to compare the difference between each mouse line (WT, HDAC6 KO and HDAC6 ZnFm) at each timepoint. p values between KO and WT, or ZnFm and WT are presented as: ∗p < 0.05; ∗∗p < 0.01; ∗∗∗∗p < 0.0001; ns, no significant difference. Supporting Figure S2: A, immunoblot of HDAC1, HDAC2 and HDAC6 protein level in WT THP-1 cells vs DMSO or dTAG treated F10-FKBPF36V THP-1 cells. DARPin F10 was detected by anti-HA antibody. B, FKBPF36V THP-1 cells were established for evaluating the possible effect of dTAG on IL-1β release. Cells were first treated with PMA (0.5 μM) for 3 hr, then medium was changed and vehicle or dTAG was added (1 μM), followed by 20 μM nigericin for 3 hr. Supporting Figure S3: A, CTG cell viability assay to analyze degrader XY-07-035 toxicity in HEK293T, Jurkat and U937 cells. Cells were treated with different concentrations of the degrader, and cell viability determined by CTG assay was normalized to the non-treated group. Scale bar is presented as mean ±SD. B, THP-1 cells, with or without in vitro differentiation, were incubated with XY-07-35 and the HDAC6 level was examined by immunoblotting. Supporting Figure S4: Cell viability was measured by resazurin assay, and fluorescence intensity was a representative of the viability. No significant difference was found among DMSO, XY-07-35 and XY-07-191 groups. Maximum survival was obtained when cells were treated with the inflammasome inhibitor CGP. [file mmc1.pdf]
